# Instructional Design Strategies for Teaching the Mental Status Examination and Psychiatric Interview: a Scoping Review

**DOI:** 10.1007/s40596-022-01617-0

**Published:** 2022-03-22

**Authors:** Eric Lenouvel, Camelia Chivu, Janet Mattson, John Q. Young, Stefan Klöppel, Severin Pinilla

**Affiliations:** 1 University Hospital of Old Age Psychiatry and Psychotherapy, Bern, BE Switzerland; 2 Karolinska Institute, Solna, Stockholm, Sweden; 3 Zucker School of Medicine at Hofstra/Northwell, Hempstead, NY USA; 4 Zucker Hillside Hospital at Northwell Health, Glen Oaks, NY USA; 5 Institute for Medical Education, Bern, BE Switzerland

**Keywords:** Psychiatry, Mental status examination, Clinical interview, Cognitive task analysis, Instructional design

## Abstract

**Objective:**

The psychiatric mental status examination is a fundamental aspect of the psychiatric clinical interview. However, despite its importance, little emphasis has been given to evidence-based instructional design. Therefore, this review summarizes the literature from an instructional design perspective with the aim of uncovering design strategies that have been used for teaching the psychiatric interview and mental status examination to health professionals.

**Methods:**

The authors conducted a scoping review. Multiple databases, reference lists, and the gray literature were searched for relevant publications across educational levels and professions. A cognitive task analysis and an instructional design framework was used to summarize and chart the findings.

**Results:**

A total of 61 articles from 17 countries in six disciplines and three educational levels were identified for data extraction and analysis. Most studies were from the USA, presented as educational case reports, and carried out in undergraduate education in the field of psychiatry. Few articles described the instructional rationale for their curriculum. None of the studies compared the effectiveness of different instructional design components. Reported learning activities for each task domain (knowledge, skills, and attitudes) and for each step of an instructional design process were charted. Most articles reported the use of introductory seminars or lectures in combination with digital learning material (videos and virtual patients in more recent publications) and role-play exercises.

**Conclusions:**

Educators in psychiatry should consider all task domains of the psychiatric interview and mental status examination. Currently, there is a lack of empirical research on expertise acquisition and use of instructional design frameworks in this context.

Since the first systematic work on psychopathology was done by German psychiatrist and philosopher Karl Jaspers in 1913 [1], administering the mental status examination (MSE) has become a core clinical skill. The first use of the term ‘mental status examination’ has been attributed to the Swiss-American psychiatrist Adolf Meyer [2]. Medical students have to master it based on competency-based undergraduate curricula [3]. The psychiatric interview, including the MSE, is a clinical assessment of cognitive, emotional, and behavioral functioning [4–8]. While memorizing the definitions of psychopathological symptoms and a corresponding sequence of questions may appear to be a straightforward exercise, conducting the MSE in a clinical interview can become an overwhelmingly complex clinical task—especially for novice trainees. This complexity arises from having to conduct the MSE while concurrently easing a patient’s suffering, building a therapeutic alliance, obtaining a psychiatric database, interviewing for a diagnosis, and negotiating a treatment plan under sometimes challenging and diverse conditions [4, 5]. Despite an abundance of literature on each of these elements [4–9], and on more general communication skills, evidence and best practice recommendations on instructional design for teaching the MSE as part of the psychiatric interview remain scarce. The authors found only one review that explored quantitative evidence for effectively teaching the MSE, and it highlighted the need for a broader review of instructional methods [10].

Instructional design development typically starts with an analysis of the desired learning outcomes, which, in competency-based medical education, are also termed “whole-tasks,” “complex tasks,” or “entrustable professional activities” (EPAs) [3, 11]. To describe such a task from an expert’s perspective, educators have used cognitive task analysis (CTA) to analyze and structure the relevant knowledge, skills, and attitudes of complex tasks in a range of disciplines—including medicine [12]. Research in other educational fields has shown that instructional designs based on CTA have resulted in a 31–46% post-training performance gain [12]. The next step in instructional design development is to select and orchestrate specific instructional strategies and sequences. This can be done using the four-component instructional design (4C/ID) model [11], which is based on learning tasks (*component 1*), supportive information (*component 2*), procedural information (*component 3*), and part-task practice (*component 4*) [11]. To our knowledge, CTA and the 4C/ID have not been used for reviewing the educational literature on teaching the MSE as part of a psychiatric interview.

Effective use of the MSE in a psychiatric interview is important not only for psychiatrists. Mental disorders are highly prevalent across health service sectors [13]. An estimated 25% of family practice consultations are for psychiatric reasons, and 26–33% of the population suffers from a common psychiatric disorder at some point in their lives [13, 14]. The chronology of psychiatric disorders has been shown to be longer than that of somatic disorders, and is associated with significant societal costs. Consequently, effective instructional designs for teaching how to assess psychiatric disorders are relevant for a wide range of medical educators and clinical professions.

The aim of this study was to review the literature and synthesize current expert knowledge on the task domains of the MSE as part of the psychiatric interview and to extract information on instructional components. These findings could serve both educators involved in designing longitudinal curricula for clinical interviewing skills of health professionals as well as clinical educators involved in workplace-based curriculum design. A comprehensive understanding of the task domains of the MSE will allow for targeted and situated educational interventions and inform future research in this area.

## Methods

The authors conducted a scoping review of the medical education literature and followed the scoping review guidelines of Arksey and O’Malley to identify relevant articles [15]. The research question was: “What instructional design strategies for teaching the psychiatric interview and MSE have been used or studied in the education of health professionals?” For charting the data and collating and summarizing the results, both a cognitive task analysis (CTA) [12] and the four-component instructional design (4C/ID) [11, 16] approach were used to derive instructional design implications and identify research gaps.

### Data sources and Search Strategy

The following search terms and Boolean operators were used: “*Mental Status Exam,”* OR “*Mental Status Examination” AND (scholarship OR education OR teaching OR learning OR pedagogy).* The search queries were selected based on the MSE commonly being called an exam or examination and paired with synonyms of pedagogic terms. The following databases were searched: Ovid, MEDLINE, PsycINFO, Embase, ERIC, MedEdPortal, the archives of Academic Psychiatry, Networked Digital Library of Theses and Dissertations, standard textbooks, the gray literature (e.g., materials and documents that are typically not published in academic journals such as educational material or technical reports), Google scholar (first ten pages), Association of Directors of Medical Student Education in Psychiatry (ADMSEP) resources, and personal files. Only peer reviewed, published journal articles in the English, French, or German languages were considered. The last date search was performed on the PubMed and Academic Psychiatry archives on August 23, 2021.

### Screening and Selection of Articles

Article titles and abstracts were screened by two authors independently. Articles published since 1970 with direct relevance to teaching content and/or the process of the MSE as part of the psychiatric interview in any clinical health profession were included. Exclusion criteria were as follows: articles that omitted mention of the psychiatric interview (including the MSE) in either the title, abstract, or content, and articles that omitted discussions on teaching methodology. Of special note, the Folstein Mini Mental State Examination was not specifically explored as the authors considered it to be out of scope of this review. Following the recommendations for scoping reviews, article reference lists were searched in duplicate by two of the authors for relevant articles [15]. Their conclusions were then compared for variations and discussed with a third author. Sources of evaluative bias were identified through duplicate analysis. Any discrepancies were discussed between authors until a consensus was established.

### Data Extraction, Analysis, and Synthesis of Results

Included articles were initially charted in an Excel table and information regarding educational intervention (medical specialty, educational level, reported educational outcomes, task domains, learning tasks and, if available, instructional design) were extracted. Each article’s extractions were discussed and are synthesized in Table 1 (task domains and corresponding instructional strategies) and Table 2 (instructional design components). We focused on the first of five stages of evidence-based CTA approaches. This involved the identification of target performance goals and a review of general knowledge about task domains to chart relevant terms and processes [12]. In a second step, we used the 4C/ID model by Van Merriënboer [11] to organize, interpret, and synthesize the extracted data according to learning tasks (*component 1*: authentic professional tasks), supportive information (*component 2*: how to approach the tasks and how the domain is organized), procedural information (*component 3*: describing step-by-step procedures to perform routine aspects of the tasks), and part-task practice (*component 4*: repetition of aspects that need to be automated). An instructional design model for the MSE is depicted in Table 2.

**Table 1:** Task domains and corresponding instructional strategies of the mental status examination (MSE) as part of a psychiatric interview

Domain^a^	Domain specification^b^	Instructional strategies
Knowledge	• Content of the MSE and psychiatric interview • Structure/organization/process of a psychiatric interview in different clinical situations • Standards for documentation of the MSE • Diagnostic criteria for mental diseases • Knowledge of communication techniques including for challenging situations • Identification and assessment of agitation • Mental/conceptual models for structuring the psychiatric interview or synthesizing findings from the MSE during a psychiatric interview	• Didactic seminar/lecture participation [17–29] • Watching clinical interview video libraries with expert performances [2, 17, 19, 23, 30–37] • Working through virtual patient scenarios [38–44] • Knowledge tests [17, 23, 25, 44–46] • Familiarization with documentation templates/checklists (print or electronic) [18, 47–49] • Facilitated small group sessions [27, 46, 50–52] • Teaching resident rotation (preparation of materials) [26, 50, 53] • Readings (book chapters, articles) [21, 51, 52] • Written (homework) exercises [25, 29] • Familiarization with mental and conceptual models of psychiatric interviewing [2, 29] • Watching selected movies depicting psychiatric disorders [54] • Self-regulated practice with peers [51]
Skills	• Communication skills • Establishing the physician-patient relationship • Non-verbal skills • Observational skills • Psychotherapeutic mini-intervention skills • Regulation of one’s own emotions • Focused exploration skills based on differential diagnosis (including clinical reasoning skills) • Verbal de-escalation skills • Personal safety and escape skills	• Role-play exercises [2, 24, 25, 27, 29, 32, 43, 46, 51, 52, 55–63] • Directly/indirectly observed sequences with real patients in in-/outpatient settings with expert feedback in individual or group learning settings [18, 21, 28, 64–69] • Clinical rotations/attachments/clerkships [33, 34, 45, 48, 51, 52, 69, 70] • Practice with standardized interview guides or documentation templates [22, 47, 48, 71] • (Video-taped) interactions in person or virtually with real/simulated or manikin patients [23, 32–35, 46, 55, 69, 70, 72] • Teaching resident rotation (supervising first year residents/medical students) [26, 50, 53] • Microtraining for specific interviewing skills [2, 21] • Mentoring program [21]
Attitudes	• Empathy • Autognosis/self-knowledge acquisition • Reflection of transference and countertransference • Developing awareness of world views and personal value systems • Congruence • Awareness of the level of regard • Developing cultural awareness	• Direct/indirect observation/supervision of learners’ real patient interactions [2, 21, 58, 64] • Clinical rotations/attachments/clerkships [51] • Students playing patient roles [60] • Balint groups for students [73] • Process rounds [28] • Reflection exercises [74]

^a^Domains were derived from elements that constitute complex learning as described by Vandewaetere et al. [16]

^b^An extensive list with formulated learning goals for each domain can be found in the appendix of Shea et al. [21]

**Table 2:** Data extraction using an instructional design model for the task “Conducting a psychiatric interview including a mental status examination” [11, 16]

Main steps	Instructional principles for psychiatric interview and MSE	Selected examples from reviewed articles (should be selected based on local context)
Component 1: Steps 1–3 (Learning tasks)		
1. Design learning tasks	- Representative psychiatric cases - Variation in complexity - Adjusted to educational phase (preclinical, clerkship year, first-year resident etc.) - Should include routine and non-routine aspects - Variation in instruction	- See learning tasks identified for knowledge domain in Table 1 - Representative psychiatric consultation situations (clinical presentations, e.g., based on frequency of presentation) [33, 36] - Routine: Psychiatric interview and MSE with cooperative, calm patient [32] - Non-routine: acute suicidality, agitated behavior, e.g., role-play exercises [61]
2. Develop assessment instruments	- Assessment instruments serve for quality assurance of clinical standards - Whole-task versus part-task assessments - Assessment matrix* - Provide worked examples of expected standards	- Peer-observation checklist [18] - Standardized documentation forms [4, 32, 71] - Knowledge testing for elements of psychiatric interview and MSE [36] - Worked examples as supportive information to understand interview processes [21] - Workplace-based assessments [66]
3. Sequence learning tasks	- Optimize learning process with sequencing from low to high complexity - With decreasing support/supervision	- Example for outpatient clinic in a workplace-based curriculum with 50-min consultations with focus on diagnosis and triage can be found in Shea et al. [21] - Sequence example for a preclinical introduction curriculum in Martin et al. [36] - Using the Kolb-cycle for sequencing learning activities [69] - Currently there is no specific evidence for sequencing order recommendations available (e.g., theory -> video-based -> role-play -> simulated patient encounter -> direct and indirect observation with real patients)
Component 2: Steps 4–7 (Supportive information)		
4. Design supportive information	- Information material for learners to complete routine and non-routine aspects of task classes. - To identify or develop supportive information, analyze relevant cognitive strategies and mental models	- Provide performance objectives/learning goals [21] and performance standards (e.g., checklists or interactive videos) [32, 36, 71] - Define essential and optional readings - Offer cognitive feedback on students’ task performance (provide expert answers for tasks and present problem solving approaches where meaningful) [66]
5. Analyze cognitive strategies	Provide systematic approaches for problem solving (e.g., being critically aware of heuristics and rules-of-thumb in clinical reasoning)	- Use of schematic outlining of interview process, e.g., as described for the “facilics” model [66] - Include teaching sessions for specific interviewing strategies (e.g., for suicidality exploration) [57] - Identify heuristics for interviewing or clinical reasoning when performing the MSE such as [4, 28]: catch-all question/miracle question*, complementary shift, diffusion statements etc.
6. Analyze mental models	Personal theories of trainees regarding mental illness and their treatment that are created and continuously developed with increasing expertise	- Provide scaffolding for personal illness scripts (to form mental models of prototypical interview flow for frequent clinical presentations) [29, 66] - Systematic indirect/direct supervision [66, 73]
Component 3: Steps 7–9 (Procedural information)		
7. Design procedural information	Information on how to perform recurrent aspects of learning tasks, ideally provided as just-in-time information, gradually decreasing with students’ learning progress. If material is already available, this can be used (e.g., checklists, quick aids etc.); if not, steps 8 and 9 help with developing new material	- Just-in-time procedural information can be provided for novice learners in virtual patient scenarios [40, 41, 44] - Direct supervision in role-play exercises [60] - Direct supervision in early clinical experiences through assigned mentors or teaching faculty [21, 50]
8. Analyze cognitive rules	Identify the condition-action pairs that drive routine behavior in performing the psychiatric interview and mental status exam (e.g., in the context of assessing safety before starting the interview and arranging corresponding safe interviewing environments)	- Identify verbal and non-verbal cues that can be used both for interview structure and clinical reasoning [4] - Support students to identify communication strategies for different clinical situations (e.g., anxious or agitated patient, use of voice modulation, personal space, positioning) [18]
9. Analyze prerequisite knowledge	What knowledge is necessary to be able to apply the cognitive rules	- See knowledge task domain in Table 1 - Knowledge of general and specific clinical interviewing principles [4]
Component 4: Step 10 (Part-task practice)		
10. Design part-task practice	Compilation and strengthening of rules, e.g., practicing “spot diagnoses” (i.e., quickly formulating main differential diagnoses after a first clinical impression) with prototypical clinical presentations	- Training with video-databanks (in particular novices), self-testing of knowledge (symptom definitions, diagnostic criteria etc.) [23, 25, 36] - Content and process-specific practice sessions [28, 73]

*Assessment matrix: an overview of frequencies and types of assessments used in a program

Example for catch-all question as interviewing strategy: “We’ve talked about a lot of things that your partner is upset about with you; are there any we haven’t talked about yet?” adapted from [4]

## Results

### Included Articles

The initial search yielded 4,152 articles. After the removal of duplicates and an independent screening of titles and abstracts by two researchers, the full texts of 101 articles were assessed. In total, 61 articles [2, 10, 17–76] were used for the data extraction. In addition, six standard psychiatry and psychiatric interviewing textbooks or chapters focusing on the MSE, psychiatric interviewing, and instructional design were identified and screened [4–9].

The characteristics and main aspects of the included articles from 17 different countries were summarized descriptively. Most studies were from the USA (*n*=23.4%), the UK (*n*=5.8%), and Australia (*n*=3.5%) but also from South American and Asian countries. Prevalent study types were educational case reports (*n*=17.3%), cohort studies (*n*=9.2%), and expert consensus reports (*n*=6.1%) and less often randomized-controlled trials (*n*=4.7%) and only one ethnographic study. The studies were typically carried out in psychiatry (*n*=52.9%), primary care (*n*=6.1%), and nursing (*n*=5.8%); however, we also found studies in the context of emergency medicine and psychosomatic medicine. In terms of educational level, *n*=39.6% of the included studies were carried out at the undergraduate level, *n*=24.4% on the graduate level, and *n*=3.5% on the continuing education level. While most studies reported educational outcomes in terms of student satisfaction ratings (*n*=22.4%), observer-based performance ratings (*n*=14.2%), and self-assessment of competence (*n*=7.1%), a large proportion did not report any educational outcome measures (*n*=17.3%).

The identified main structures for written MSE reporting were juxtaposed with the currently taught MSE reporting structure at our institution and differed only in terms of sequence and level of detail but contained the same elements of psychopathology.

### Cognitive Task Analysis

Table 1 depicts the extracted and synthesized relevant knowledge, skills, and attitudes for conducting the MSE during a psychiatric clinical interview. For each domain (knowledge, skills, and attitudes), the reported learning tasks used in the studies were also extracted. Typically, these learning tasks were described as part of an educational intervention and in the context of a set of tasks without explicit reference to learning goals or task domains. However, we were able to identify several discrete learning tasks that could be rearranged and sequenced if needed.

Most articles reported the use of introductory seminars or lectures for teaching the knowledge domain of psychiatric interviews and the MSE in combination with knowledge tests and digital learning material. The latter included expert role modeling in video-recorded interviews and, in more recent publications, different types of virtual patient scenarios (Table 1). To teach clinical interviewing, educators used Virtual Human Agent technology [38], virtual reality scenarios [39], virtual patient programs [40, 41], illness-specific virtual patient systems [42], teaching suicide risk assessment with a virtual patient [43], and a virtual clinic simulator [44]. Moreover, the search yielded role-play exercises as the most frequently reported learning task for different aspects of the skills domain (see Table 1 for specific references). To practice the psychiatric interview and the MSE, educators used actors, clinical experts, more advanced trainees, peer students, or avatars as standardized, simulated, or virtual patients for students.

Another frequently used method was need-based or longitudinally direct or indirect supervision with expert clinicians who provided formative feedback for developing interviewing and examination skills while learners saw real patients. The attitude domain was the least often reported domain in educational interventions and effectiveness evaluations. Depending on the programs’ educational goals, clinical supervisors were the main source of feedback for various domains, which targeted developing an empathic attitude, acquiring self-knowledge, reflecting transference and countertransference, and critical awareness of stigma or prejudices in the context of different world views and personal value systems (see Table 1 for specific references).

Only one systematic review explored the comparative effectiveness of teaching methods for the MSE [10]. The authors reported that non-traditional teaching methods (video-taped interviews, virtual simulations, and standardized patients) tended to achieve higher student satisfaction ratings and better learning outcomes (knowledge and skills) compared to lectures and reading material alone. Due to the heterogeneity of the interventions and outcome parameters, no meta-analysis was performed. Furthermore, no studies were found that compared educational interventions such as lectures or seminars to role play, interview guides, or clinical rotations alone or in different combinations or sequences.

### Instructional Strategies

Instructional strategies relevant for developing a complex task course based on the four-component instructional design (4C/ID) model [16] were charted according to the ten steps of the instructional model (Table 2). Only a few articles described the instructional rationale for their curriculum in detail [21, 26, 29, 36, 51, 69]. The most elaborated curriculum was a 2-month, workplace-based psychiatric interview program based on previously published literature and expert opinion by Shea and Mezzich [2]. This article outlined concrete learning goals and a sequence and combination of theoretical training, experiential and self-regulated learning, and direct and indirect supervision. While the educational outcomes were measured only at the learners’ satisfaction level (with overall high satisfaction levels), no comparison with other instructional strategies or sequences was made. The feasibility of implementing such a program in multiple sites was not investigated.

## Discussion

In this scoping review, the authors searched the literature for articles on instructional designs for teaching the MSE as part of the psychiatric interview using both a cognitive task analysis (CTA) and a four-component instructional design (4C/ID) model framework. The key result of this review is the synthesis of specific knowledge, skills, and attitudes regarding the MSE within the psychiatric interview and the corresponding instructional design strategies that have been implemented or studied. Educators and researchers could use this as a framework for local curriculum development or educational design studies.

The large body of literature found for this review illustrates that teaching and learning the MSE under workplace-based curricula cannot be separated from conducting a clinical (psychiatric) interview. Therefore, the MSE should not be considered exclusively as a “technical” skill comparable to a physical examination, as suggested by some authors [8]. Instead, it is one element of a specific form of the clinical interview that relies on general interviewing and communication competencies that all physicians need when interacting with patients [4]. For example, end-of-undergraduate-training EPAs in the USA do not have a separate EPA for the MSE. Instead, it is nested within larger activities such as a diagnostic interview, follow-up visit, or emergency management [3]. Only a few articles attempted to address the complex nature of teaching this competence comprehensively, typically at the cost of empirical research designs [21, 57, 61, 66]. The number and breadth of studies (both qualitative and quantitative papers on relevant instructional designs for all task domains of the MSE) included in this review exceeds those that were included in a previous systematic review by Xie and colleagues [10], which was limited to quantitative studies. Specifically, over 15 relevant papers have been published since the Xie et al. review, many of which have used innovative digital instructional designs.

Earlier studies [10, 17, 46] have provided evidence that experiential forms of learning the psychiatric interview and MSE (i.e., through role play and practice with standardized patients) in comparison to didactic teaching methods alone (i.e., lectures and readings) tend to lead to better educational outcomes. This is not surprising in the era of competency-based education. However, even well-designed and more recent studies only marginally describe the rationale for their instructional design (i.e., choice and sequencing of instructional methods) or not at all [25, 36]. There is also a lack of studies investigating what combination of instructional design elements is the most effective for each educational stage on the novice-expert-continuum (pre-clinical student versus clerkship student versus first-year resident versus specialist). Therefore, the findings of this review can provide educators with instructional strategies that take into account each task domain of the MSE and, hence, future research can address questions of sequencing and adjustment for the expertise level of trainees.

Technology-enhanced learning such as annotated or interactive clinical video databases or virtual patient scenarios appear to be cost-effective options (especially for novice learners) and can help to ensure that all learners are exposed to the same range of clinical presentations independent of the clinical context of a clerkship rotation [2, 17, 19, 23, 30–32, 36, 38–44]. This is also relevant from a patient safety perspective as it ensures that every student develops basic competencies before seeing real patients. In one randomized-controlled trial, there was no significant learning performance differences when comparing video-based and virtual patient scenarios, but students preferred video-based instruction [43]. Similar study designs that compare variations of the same instructional method (e.g., role-play with versus without specific introductions to this learning method [61]) would be helpful for evidence-based instructional design recommendations. The details of an instructional design might have an impact on the acceptance of the learning method and transfer of acquired knowledge into clinical practice. Given the challenges with the authenticity of simulated clinical interviews, learner anxiety over performing a clinical interview in front of an audience, and the potential lack of confidence in these instructional methods [43, 61], it may be worthwhile to explore the added value of investing time and resources in evidence-based instructional design. An emerging area of educational research is the use of digital learning environments for teaching the MSE and psychiatric interview (e.g., virtual patient scenarios). The instructional design formats identified in this scoping review might inform future research questions and study designs.

This scoping review has several limitations. First, while studies from 17 different countries were included, this was a non-representative sample. Psychiatry as discipline is deeply embedded in the linguistic and cultural context where it is practiced. Therefore, important perspectives on teaching and learning the clinical psychiatric interview and MSE in other countries and regions may have been missed. In addition, successfully implemented educational material and concepts might not always be translated and published in scholarly journals. Moreover, academic institutions might not systematically support the scholarship of teaching and learning the MSE and the clinical psychiatric interview even though this would be necessary for sharing knowledge and advancing the field. Second, the authors’ own biases may have affected how these records were perceived as all of the authors work in the field of health care. Non-medical professionals or patients might have made different determinations. Third, under-researched teaching and learning situations in the context of the psychiatric interview and MSE such as accounting for cultural diversity, using digital interviewing formats, interviewing geriatric patients, or forensic psychiatric interviewing were not represented in this review and warrant further attention.

In conclusion, educators involved in the design of MSE curricula and the psychiatric interview—or in longitudinal communication curricula—should consider all the task domains of the psychiatric interview early in undergraduate training and deliberate on instructional design and the potential synergies between disciplines. While not all identified task domains can be covered in short clerkship rotations, it seems worthwhile to invest time in understanding which task domains of the psychiatric interview might already be covered in other communication skills trainings (e.g., in pre-clinical sessions or breaking-bad news training in other medical disciplines), and which not. Ideally, a psychiatry clerkship curriculum would build on and inform longitudinal communication curricula involving other disciplines or residency programs. The four-component instructional design model can be used to identify potential gaps in a given curriculum and lead to adding or adjusting instructional methods.

While a broad range of instructional methods has been used in various educational contexts for teaching the psychiatric interview and MSE, no clear standard for an instructional design has emerged. We suggest using the available evidence for developing instructional design blueprints for teaching the psychiatric interview. Future research should explore facets of expertise acquisition in conducting the psychiatric interview and the educational benefit of novel digital learning environments in this context.
